# Bridging the gap: aligning physical work capacity testing with actual endurance performance in military settings

**DOI:** 10.3389/fpsyg.2025.1536197

**Published:** 2025-01-27

**Authors:** Jan Drozd, Jiří Neubauer, Jiří Sekanina, Marek Sedlačík

**Affiliations:** ^1^Department of Military Science Theory, University of Defence, Brno, Czechia; ^2^Department of Quantitative Methods, University of Defence, Brno, Czechia; ^3^Physical Training and Sport Centre, University of Defence, Brno, Czechia

**Keywords:** PWC 170, Cooper test, 12-min run, physical fitness, recruitment, endurance, prediction

## Abstract

Physical fitness tests are essential for evaluating the endurance capabilities of military personnel. In many armed forces, including the Czech Republic’s, the Physical Working Capacity at a heart rate of 170 beats per minute (PWC 170) test is used to predict performance on the 12-min Cooper run, a widely accepted measure of endurance. However, concerns exist regarding the accuracy of the currently used conversion between PWC 170 test results, specifically W170/kg (watts per kilogram of body weight), and actual 12-min run performance. This study directly investigates the relationship between W170/kg and 12-min run times among military recruits and students (military personnel) from a university with a military program. Utilizing regression analysis, we found a significant positive correlation between W170/kg and 12-min run performance. However, the currently used conversion significantly underestimated actual 12-min run performance across all analyzed groups. These findings highlight a critical need to revise the existing conversion standard between W170/kg and 12-min run performance to ensure a more accurate and effective assessment of endurance capabilities in military personnel.

## 1 Introduction

Military personnel are expected to perform physically demanding tasks under challenging conditions. A high level of physical fitness is therefore essential for operational effectiveness, reducing injury risk, and ensuring the overall wellbeing of soldiers. To assess and maintain these standards, armed forces worldwide employ rigorous physical fitness assessments during recruitment and throughout a soldier’s career. These assessments typically evaluate various aspects of physical capacity, including aerobic endurance, muscular strength and endurance, flexibility, and body composition (Sedlačík et al., 2023).

The Cooper test, a 12-min run designed to measure maximal aerobic endurance, is a widely recognized and implemented assessment tool in military contexts globally (Cooper, 1968). This test measures the maximum distance an individual can run in 12 min, providing a simple and practical way to estimate their aerobic fitness level. The test’s simplicity, cost-effectiveness, and ability to assess a large number of individuals simultaneously have contributed to its widespread adoption. While frequently used by the Czech Army, the 12-min run holds significance in fitness evaluations for both potential and serving soldiers in numerous other militaries as well (Maksud and Coutts, 2013).

Similarly, the Physical Working Capacity Test (PWC 170 test), often conducted on a cycle ergometer, is another common assessment tool used to gauge cardiorespiratory fitness. This graded exercise test determines an individual’s physical work capacity (W170) at a heart rate of 170 beats per minute, expressed relative to body weight (W170/kg). This metric is considered a reliable indicator of VO_2max_, which represents the maximum amount of oxygen an individual can utilize during intense exercise (Cherepov et al., 2017; Harymawan, 2020).

In fact, W170 represents the estimated power output in watts, corresponding to a heart rate of 170 beats per minute (Stork et al., 2016). For interindividual comparisons, it is recommended to adjust this power output relative to body weight [W/kg]. The obtained values indirectly indicate the degree of adaptation, primarily of the cardiovascular system, to endurance performance. A heart rate of 170 beats per minute, in a young and healthy individual, typically represents the upper limit of the circulatory system’s functional response to progressively increasing exercise intensity, where a balance can still be maintained between the energy demands of physical activity and the ability to meet those demands aerobically. At this heart rate, an optimal stroke volume is still maintained. W170 is considered a general indicator of fitness and performance for healthy untrained individuals, recreational athletes, and competitive athletes. However, this indicator is not sensitive enough for elite athletes.

The maximum amount of oxygen an individual can utilize during intense exercise VO_2max_ is a globally recognized physiological marker of aerobic fitness and endurance capacity. A higher VO_2max_ indicates a greater ability of the cardiovascular system to deliver oxygen to working muscles, translating to better performance in endurance-based activities like running, swimming, and cycling. As a key determinant of aerobic endurance, VO_2max_ is inherently linked to performance in endurance-based activities like the 12-min run (Stocker and Leo, 2020).

Recognizing the potential correlation between W170/kg and 12-min run performance, some militaries, including the Czech Army, have adopted conversion tables to predict an individual’s potential run distance based on their W170/kg score. This approach provides a convenient way to estimate endurance capabilities without requiring every individual to undergo the 12-min run, which can be physically demanding and time-consuming, especially when assessing a large pool of recruits.

However, recent studies have raised concerns about the accuracy and relevance of this conversion table in reflecting actual 12-min run performance across different populations and fitness levels (Stork et al., 2016). An inaccurate prediction tool can have significant implications for both recruitment and training programs within the armed forces. Overestimating an individual’s endurance based on W170/kg could lead to inadequate training regimens or potentially dangerous situations in operational settings. Conversely, underestimation could result in the exclusion of otherwise qualified candidates during recruitment (Stork and Novak, 2023).

This study aims to directly address these concerns by conducting a comprehensive analysis of the relationship between PWC 170 test results (W170/kg) and actual performance on the 12-min run within the context of the Czech Army. Utilizing data collected from both male and female recruits, as well as students of the University of Defence, we will employ robust statistical methods to:

- Determine the strength and nature of the correlation between W170/kg and 12-min run performance.
- Evaluate the accuracy of the existing conversion standard used by the Czech Army in predicting 12-min run distances based on W170/kg scores.
- Explore the potential need for a revised prediction model that more accurately reflects the observed relationship between these two fitness assessments.

By addressing these objectives, this study seeks to contribute valuable insights into the validity of current physical fitness assessment protocols within the Czech Army and potentially inform the development of more accurate and reliable prediction models for endurance performance across militaries globally. This, in turn, can lead to more effective recruitment strategies, tailored training programs, and ultimately, a more capable and resilient military force. However, a crucial question arises: Is the PWC 170 test truly a reliable predictor of 12-min run performance among recruits and students at the University of Defence, and how accurate is the existing conversion standard employed by the Czech Army? This study aims to address this question.

## 2 Literature review

Physical fitness stands as a cornerstone for effective military service. Soldiers must be equipped to handle demanding tasks in extreme environments, and a high level of fitness demonstrably reduces injury risk and contributes to overall combat effectiveness (Knapik et al., 2006). To assess the physical capabilities of both potential and serving personnel, armed forces worldwide employ a variety of physical fitness tests (Hauschild et al., 2017). These assessments typically target areas such as aerobic endurance, muscular strength and endurance, flexibility, and body composition.

The Cooper test, a 12-min run designed to measure maximal aerobic endurance, ranks among the most recognized and widely implemented assessments (Cooper, 1968). Its simplicity, minimal equipment requirements, and suitability for testing large groups simultaneously contribute to its widespread adoption. In many armed forces, including the Czech Army, the Cooper test holds significant weight in evaluating potential recruits (Šmíd, 2009; Ministry of Defence of the CR, 2023).

Another test frequently utilized for assessing cardiorespiratory fitness, also employed by the Czech Army, is the PWC 170 test. This graded exercise test, typically conducted on a cycle ergometer, determines an individual’s physical work capacity (W170) at a heart rate of 170 beats per minute, expressed relative to body weight (W170/kg). This metric is considered a reliable indicator of VO_2max_, a key determinant of performance in aerobic activities (Klymovych et al., 2020; Wang et al., 2023).

Given the strong correlation between VO_2max_ and performance in the 12-min run, the PWC 170 test presents itself as a potential predictor of Cooper test results. Consequently, some armed forces, including the Czech Army, employ conversion tables to estimate Cooper test performance based on PWC 170 results (Šmíd, 2009). This approach offers a way to streamline the assessment process, potentially eliminating the need for every recruit to undergo the Cooper test.

However, the accuracy of predicting Cooper test performance based solely on the PWC 170 test remains a subject of debate (Alvero-Cruz et al., 2019). Studies investigating this relationship have yielded mixed results, with prediction accuracy varying depending on the population studied, research methodology, and other factors (Kekäläinen et al., 2024). Some research indicates instances of systematic overestimation or underestimation of Cooper test performance when relying solely on PWC 170 scores (Stocker and Leo, 2020; Hauschild et al., 2017).

Clearly, predicting Cooper test performance based on the PWC 170 test represents a complex issue deserving of further exploration. To ensure the most accurate and effective evaluation of recruit fitness, it’s crucial to validate the existing conversion tables and potentially develop more precise prediction models accounting for the specific characteristics of the target population (Preti et al., 2019).

## 3 Data and methods

This study draws upon data from two distinct groups. The first group consists of 187 male Czech Army recruits, divided into two age categories: those under 30 years old (*n* = 158) and those over 30 years old (*n* = 29). These recruits represent a convenience sample, reflecting individuals undergoing standard Czech Army physical fitness assessments during their recruitment process. The second group comprises 299 cadets from the University of Defence, all under 30 years old, with 250 males and 49 females. These cadets, also active-duty soldiers, were conveniently sampled from those participating in mandatory fitness assessments within their military training program. Participants in this study were recruited through a random selection process from ongoing recruitment drives and from the student body of the University of Defence. While this represents a specific cohort, the random selection ensures that each recruit or student had an equal opportunity to be included, irrespective of their individual fitness levels or training backgrounds. This diversity within the sample reflects the reality of military settings where individuals with varying levels of experience are integrated. To ensure maximum objectivity and comparability of results, all tests were conducted under standardized conditions. These conditions were consistent for all participants and included controlled climate settings, a calibrated cycle ergometer, and standardized instructions provided before each test.

For both samples, the following data were measured:

- performance in the Cooper test (12-min run), measured as the distance covered in meters within 12 min;
- physical work capacity at a heart rate of 170 beats per minute (W170), measured in watts (W) using a cycle ergometer;
- and W170/kg, calculated by dividing W170 by the participant’s body weight in kilograms (kg).

Regression analysis, specifically linear regression, was employed to analyze the relationship between W170/kg and Cooper test performance. This method allows for modeling a linear relationship between two variables. The general form of the linear regression equation is:


$$Y_{i}=\beta_{0}+\beta_{1}x_{i}+\varepsilon_{i},i=1,2,\ldots ,n\text{,}$$

where *n* represents the sample size, *Yi* are the values of the dependent variable (performance in the 12-min run), *x_i_* are the values of the independent variable (W170/kg), *β*_0_ and *β*_1_ are the unknown real parameters (intercept and slope of the regression line, respectively), and *ε_i_* is the error term assumed to be normally distributed with a mean of zero and constant variance (Montgomery and Runger, 2011). Further analyses included: a general linear hypothesis test (Montgomery and Runger, 2011) and a test for the equality of two regression lines (King et al., 1991).

## 4 Results

This section presents the results of the analysis examining the relationship between W170/kg and performance in the 12-min run, utilizing data from both Czech Army recruits and University of Defence students.

### 4.1 Approximate conversion vs. observed data

Table 1 presents the approximate conversion standard used by the Czech Army to predict 12-min run performance based on W170/kg scores (Šmíd, 2009; Ministry of Defence of the CR, 2023).

**Table 1:** The approximate conversion standard used by the Czech Army to predict 12-min run performance based on W170/kg scores.

	Sex	Age	Unit	Comparison			
*l*_12_	Males	Under 30	Meter	2,100	2,400	2,700	3,000
W170/kg			W/kg	1.8	2.2	2.7	3.2
*l*_12_	Females	Under 30	Meter	1,600	2,100	2,400	2,700
W170/kg			W/kg	1.3	2	2.5	3
*l*_12_	Males	Over 30	Meter	1,900	2,100	2,400	2,700
W170/kg			W/kg	1.6	2.8	2.2	2.7
*l*_12_	Females	Over 30	Meter	1,400	2,100	2,100	2,400
W170/kg			W/kg	1.1	1.3	2.5	2.2

To assess the validity of this conversion standard, we first analyzed the relationship between W170/kg and 12-min run performance within each subgroup defined by sex and age as presented in Table 1: male recruits under 30 years old, male recruits over 30 years old, female cadets under 30 years old, and male cadets under 30 years old. This approach allows for a more nuanced examination of potential differences in the predictive validity of the conversion standard across these demographic categories.

### 4.2 Regression analysis

For male recruits under 30 years old, the predicted relationship based on the approximate conversion presented in Table 1 is represented by the following equation:


(1) $$l_{12}=974.5+636.6\;W170/\mathrm{kg}$$

where *l*_12_ is the distance covered in 12 min, measured in meters. The approximate conversion presented in Table 1, represented by the equation, is derived from empirical studies examining the relationship between work capacity, performance in various physical tests, and maximal oxygen consumption (VO_2max_). This conversion reflects a generalized model based on research correlating running performance, work on a cycle ergometer, and cardiovascular capacity.

Our analysis of data from 158 male recruits under 30 years old yielded a different regression equation:


(2) $$l_{12}=2005.8+250.8\;W170/\mathrm{kg}$$

This difference highlights that while the approximate conversion reflects broader physiological principles, it may not accurately represent the specific relationship between W170/kg and 12-min run performance within this particular subgroup.

**Figure 1:**
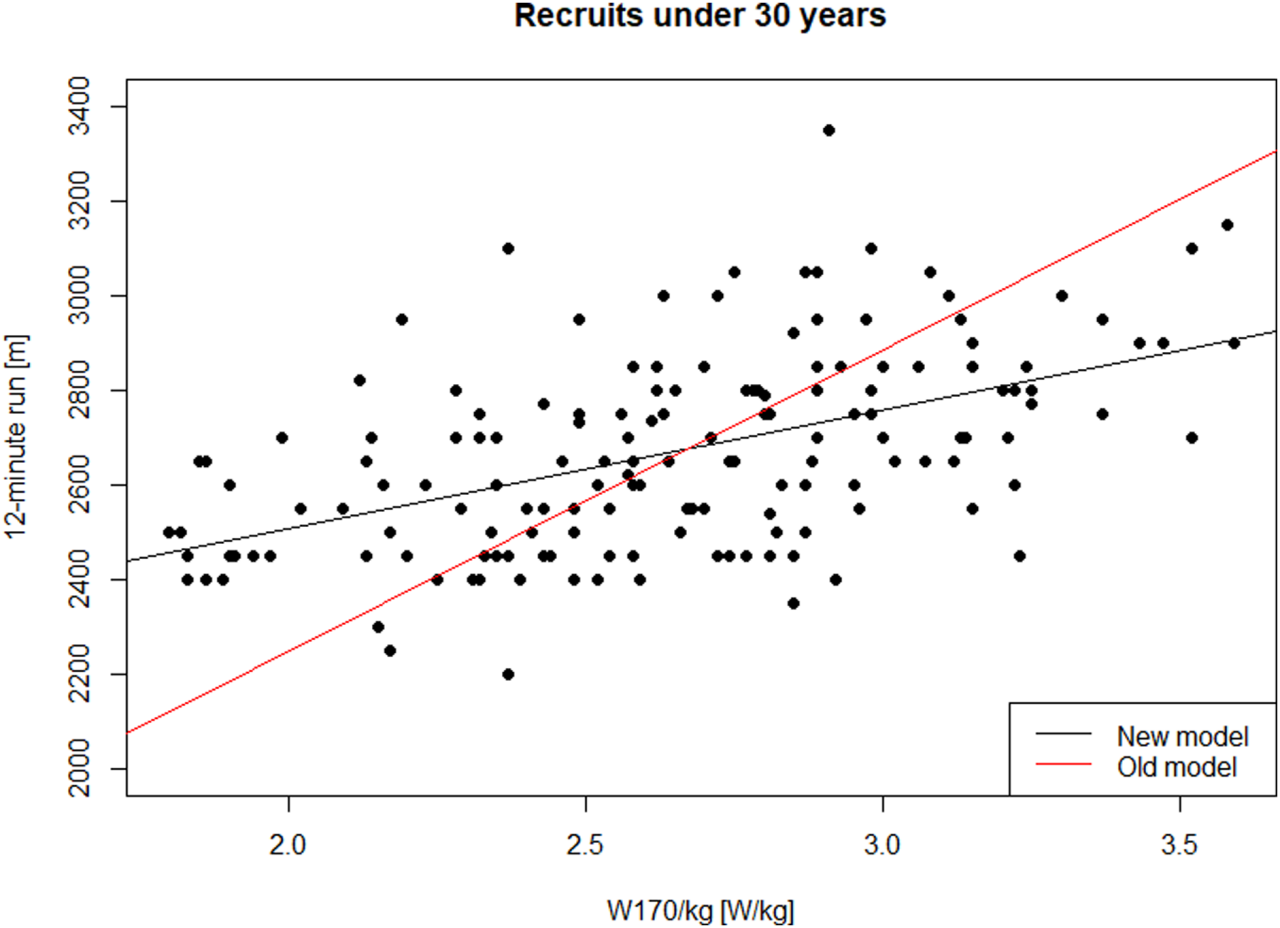
Performance in the 12-min run on W170/kg—males, recruits under 30 years of age.

This model explained approximately 26.4% of the variance in 12-min run performance (*R*^2^ = 0.264), with the slope of the regression line being statistically significant (*p* < 0.001). Figure 1 illustrates the observed data points for this subgroup, alongside the fitted regression line (Equation 2) and the line representing the approximate conversion standard (Equation 1).

A similar discrepancy was observed for male recruits over 30 years old, with the approximate conversion equation:


(3) $$l_{12}=786.6+717.3\;W170/\mathrm{kg}$$

differing significantly from the regression equation derived from our data:


(4) $$l_{12}=2301.1+104.4\;W170/\mathrm{kg}$$

**Figure 2:**
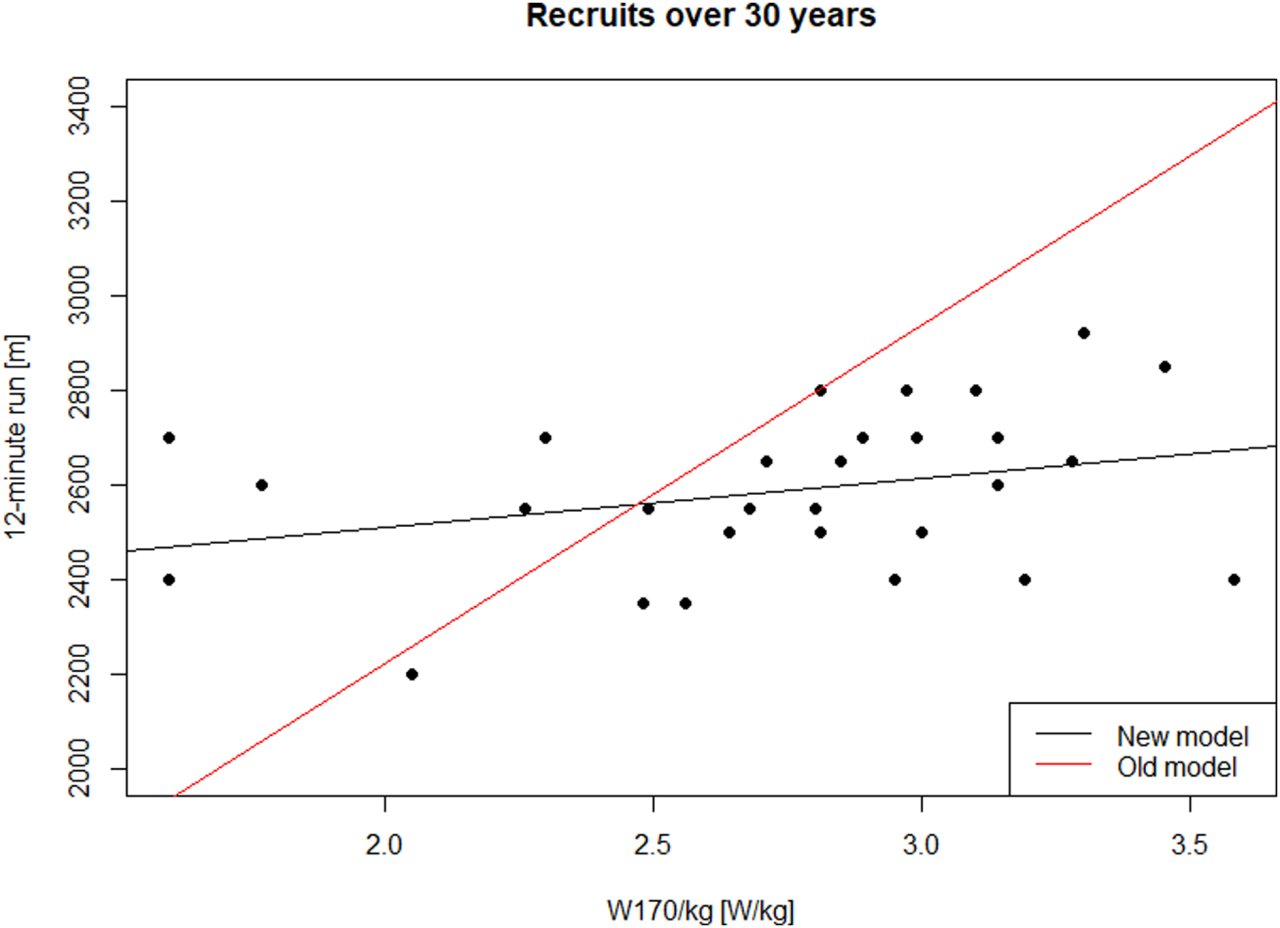
Performance in the 12-min run on W170/kg—males, recruits over 30 years of age.

The coefficient of determination (*R*^2^) of the estimated model is 0.098. The model, specifically the slope of the regression line, is not statistically significant at the *α* = 0.05 level (*p* = 0.099). Figure 2 displays the results for male recruits over 30 years old. The black line represents the trend estimated from the data (Equation 4), while the red line represents the relationship described by Equation 3 (approximate conversion). This indicates that the newly estimated relationship is significantly different (*p*-value of the test for the hypothesis that the intercept in Equation 2 equals 786.6 and the slope equals 717.3 is less than 0.001).

Next, we focus on the sample of University of Defence students, which includes 250 males and 49 females, all under 30 years old. The analysis of the male student data (*n* = 250) yielded the following regression equation:


(5) $$l_{12}=2460.1+130.2\;W170/\mathrm{kg}$$

**Figure 3:**
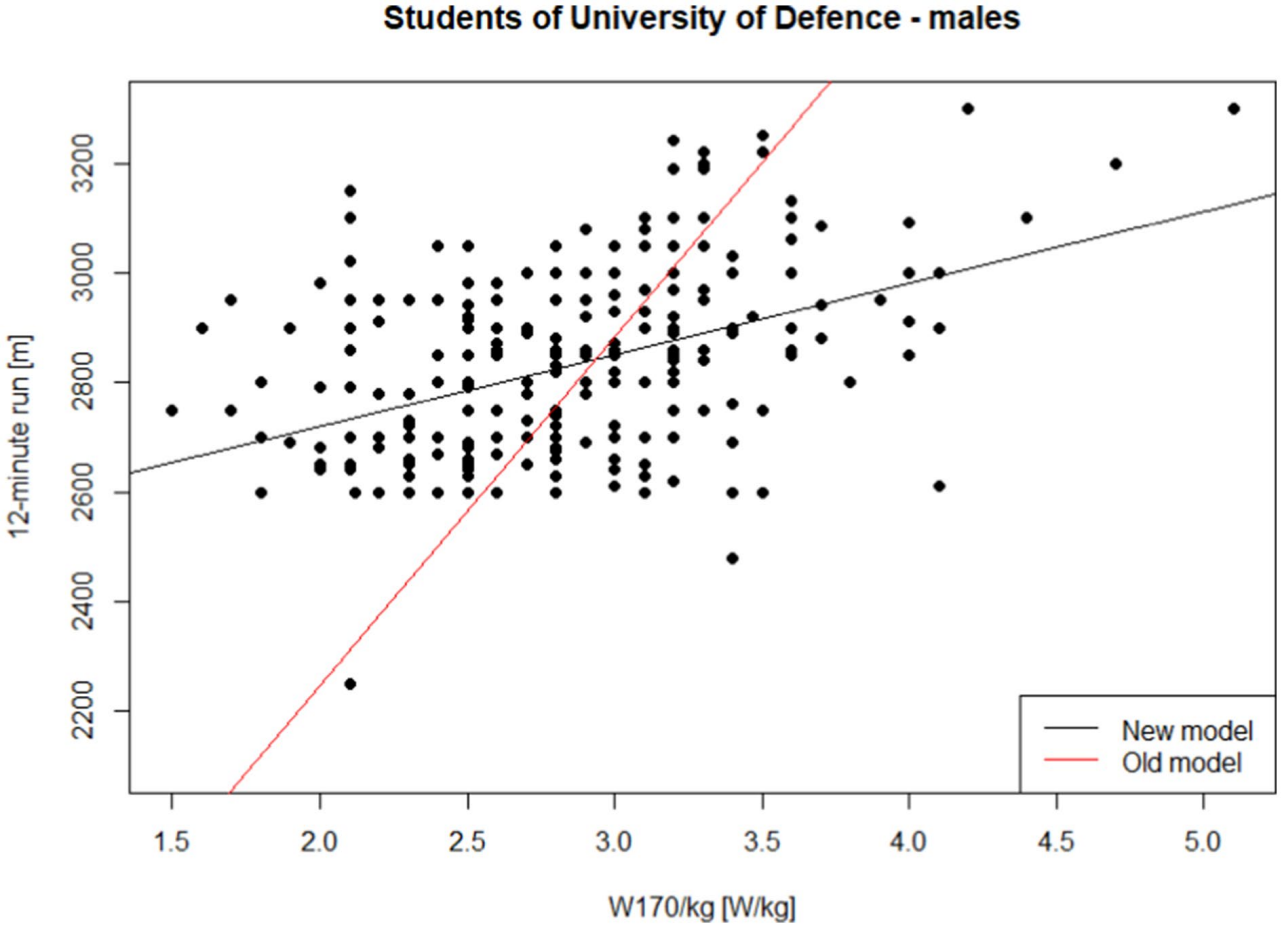
Performance in the 12-min run on W170/kg—males, students of University of Defence under 30 years of age.

The coefficient of determination (*R*^2^) for this model is 0.183. The model, specifically the slope of the regression line, is statistically significant at the *α* = 0.05 level (*p* < 0.001). Figure 3 displays the results for male University of Defence students. The black line represents the trend estimated from the data (Equation 5), while the red line shows the relationship based on the approximate conversion standard (Equation 1). Similar to previous models, the estimated relationship differs significantly from the relationship expressed by Equation 1 (*p* < 0.001).

**Figure 4:**
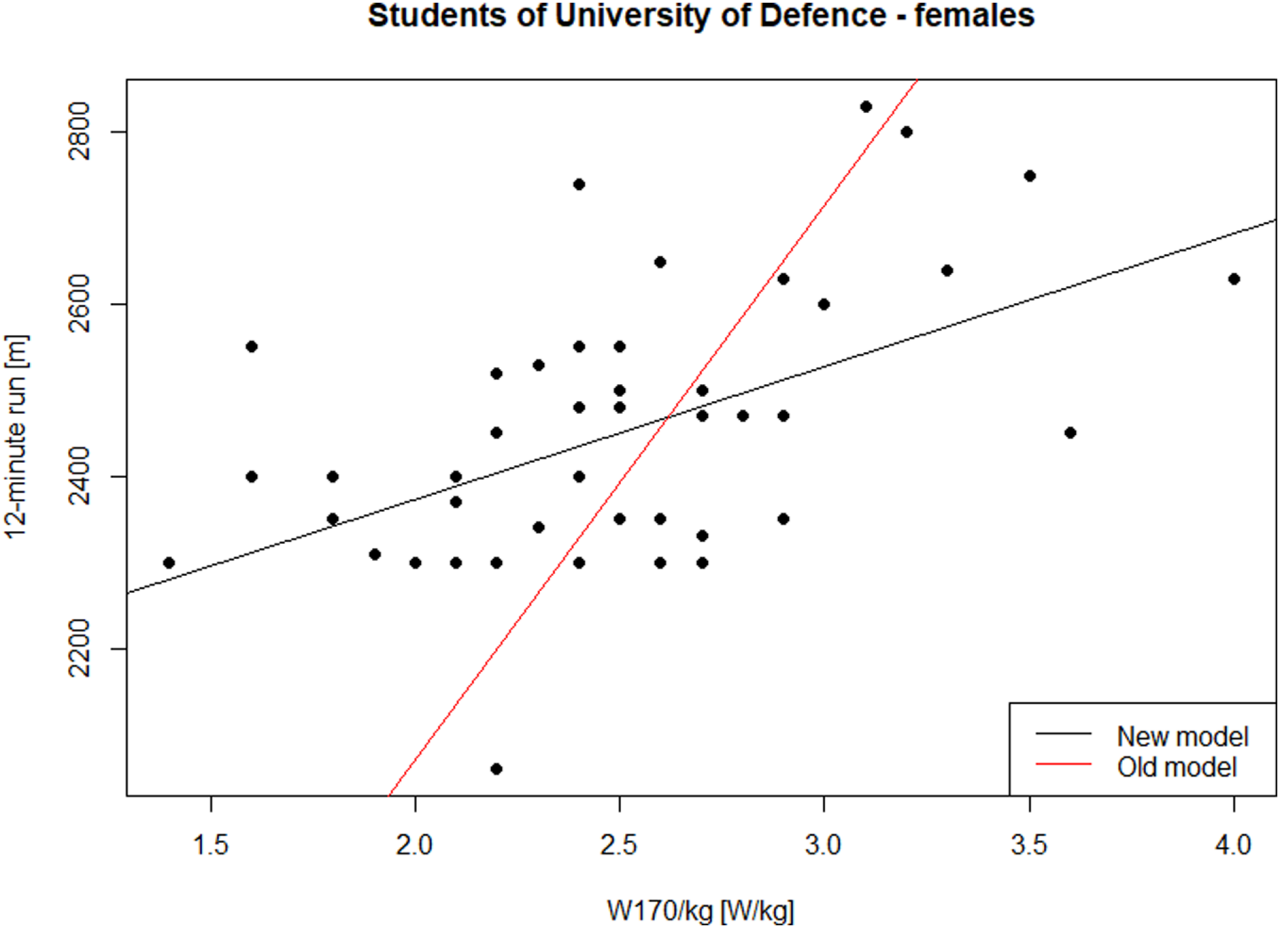
Performance in the 12-min run on W170/kg—females, students of University of Defence under 30 years of age.

Finally, we analyze the results for female students at the University of Defence. The relationship under investigation is depicted in Figure 4. For females under 30 years old, the following equation can be derived from Table 1:


(6) $$l_{12}=779.7+645.6\;W170/\mathrm{kg}$$

However, the analysis of our measured data resulted in a different estimated regression equation:


(7) $$l_{12}=2063.5+154.5\;W170/\mathrm{kg}$$

This model demonstrates a coefficient of determination (*R*^2^) of 0.265. The model, specifically its slope, is statistically significant at the *α* = 0.05 level (*p* < 0.001). Figure 4 presents the findings for female University of Defence students. The black line corresponds to the trend estimated from the data (Equation 7), while the red line illustrates the relationship based on the approximate conversion standard (Equation 6). Again, we observe a significant difference between the estimated relationship and the relationship depicted by Equation 6 (*p* < 0.001).

**Figure 5:**
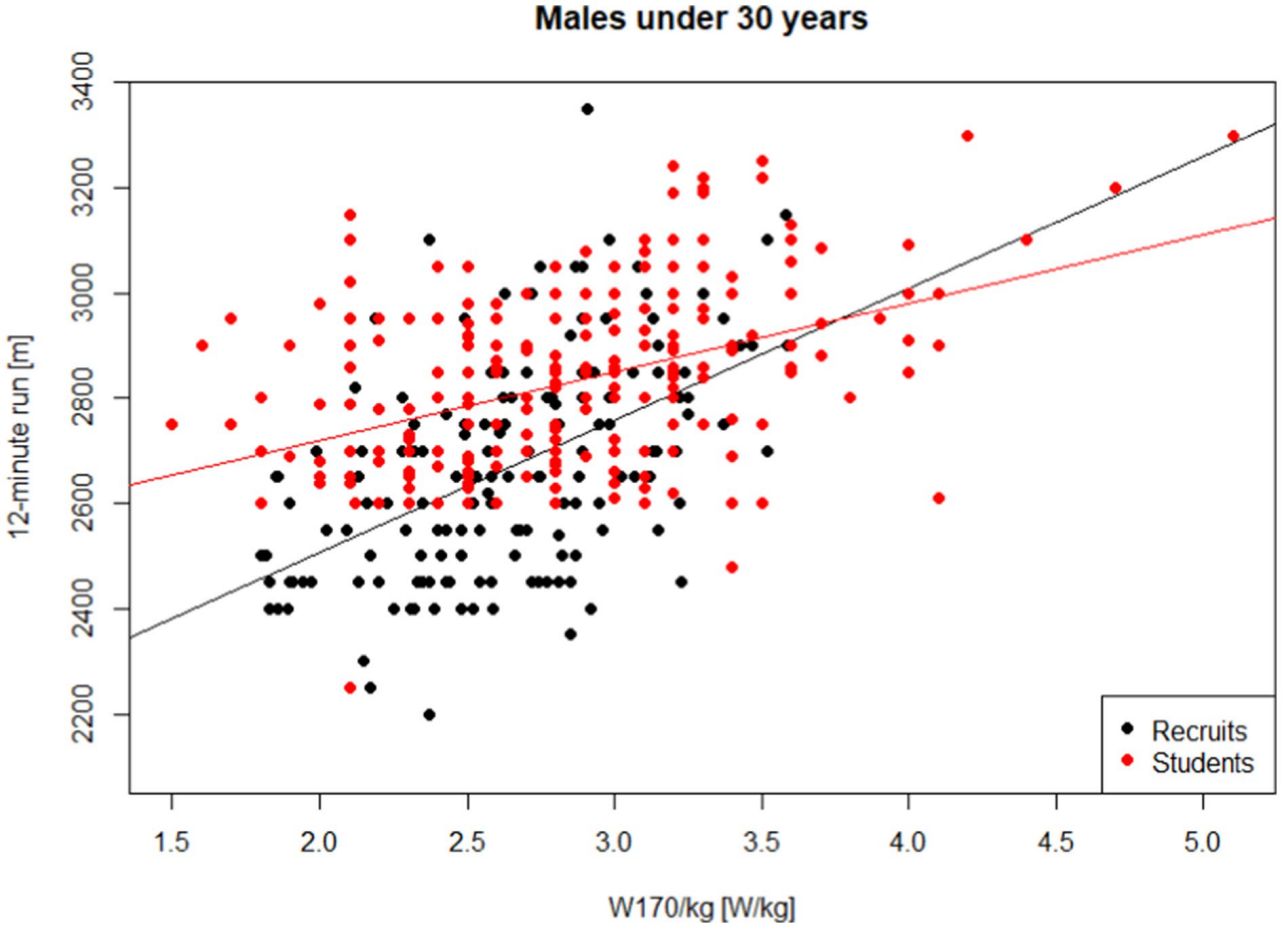
Performance in the 12-min run on W170/kg—comparison of recruits and students of University of Defence.

A pertinent question arises whether the relationships observed for recruits under 30 years old and University of Defence students are comparable. To address this, we employed a test for the equality of two regression lines (King et al., 1991). Both regression models are graphically depicted in Figure 5. Based on this test, the estimated models cannot be considered equal (*p* < 0.001).

Our analyses consistently demonstrate that the approximate conversion currently utilized to translate W170/kg scores into 12-min run performance does not accurately reflect the relationships observed in our data from both recruits and University of Defence students. Nevertheless, our analyses do confirm a significant positive correlation between measured W170/kg values and 12-min run performance. However, the specific nature of this relationship differs notably from the one suggested by the approximate conversion standard presented in Table 1.

## 5 Discussion and conclusion

This study sought to evaluate the accuracy of using the PWC 170 test as a predictor of performance in the 12-min Cooper run, a key assessment tool for Czech Army recruits. Currently, the Czech Army employs the PWC 170 test during the recruitment process, relying on a conversion standard to predict Cooper test scores. This approach stems from the logistical challenges of administering the 12-min run to a large number of potential recruits. However, as our findings demonstrate, the existing conversion standard fails to accurately reflect the observed relationship between W170/kg and actual 12-min run performance across different subgroups.

While our study shows a significant correlation between W170/kg and 12-min run performance, it also highlights the limitations of directly translating cycle ergometer performance to running performance. This discrepancy arises primarily from the distinct physiological and biomechanical demands of each activity. While the PWC 170 test primarily assesses cardiorespiratory fitness within a lower-body cycling motion, running involves a more complex interplay of muscle groups, coordination, and proprioception.

Running economy, the efficiency of energy utilization during running, plays a crucial role in this context. An individual with excellent running economy may achieve a superior 12-min run time even with a moderate W170/kg. Conversely, an individual with a high W170/kg but inefficient running biomechanics may underperform in the 12-min run.

Furthermore, biomechanical factors like limb length, muscle fiber type composition, and running technique can significantly influence running performance independent of aerobic capacity as measured by the PWC 170 test. These multifaceted aspects underscore the need for a more comprehensive understanding of the factors contributing to endurance performance in military personnel beyond isolated cardiorespiratory fitness.

This discrepancy presents a significant concern because the Cooper test holds considerable weight in the overall evaluation of potential recruits, reflecting the importance of aerobic endurance in fulfilling demanding military tasks. The inability to administer the Cooper test directly during the recruitment process necessitates a highly accurate prediction tool to ensure that individuals with sufficient aerobic capacity are selected. Overestimating endurance based on the current, flawed conversion standard could lead to recruits entering service inadequately prepared for the physical demands of training and deployment, potentially increasing injury risk and hindering operational effectiveness. Conversely, underestimation could lead to the exclusion of otherwise qualified candidates, jeopardizing recruitment goals and potentially overlooking individuals with the necessary physical capabilities.

The logistical constraints associated with conducting the Cooper test during large-scale recruitment are undeniable. However, our findings highlight the critical need for a more accurate and reliable method for predicting Cooper test performance during this phase. This need is underscored by the significant discordance we observed between the existing conversion standard and the actual relationship between W170/kg and 12-min run times. While the PWC 170 test does provide valuable insights into an individual’s aerobic capacity, its translation to run performance is complex and influenced by various factors not captured by the test itself, such as running economy, biomechanics, and pacing strategies.

This challenge underscores the need for further research into more accurate and logistically feasible methods for predicting Cooper test performance, particularly within large-scale recruitment settings. Future investigations could focus on validating Alternative Field-Based Assessments. Field tests like the Multi-stage fitness test (“beep test”) (Băiţan, 2020) and the Yo-Yo Intermittent Recovery Test offer practical advantages for mass assessments, requiring minimal equipment and efficiently evaluating aerobic capacity within a group setting (Schmitz et al., 2018). Moreover, their intermittent nature may better reflect the demands of actual military tasks compared to a cycle ergometer test. Research should prioritize validating the predictive accuracy of these tests against actual Cooper test performance.

There are also possibilities to develop integrated prediction models incorporating additional physiological metrics, such as heart rate recovery or lactate threshold, alongside data from field-based assessments, holds potential for developing more robust and accurate prediction models. Such models could provide a more comprehensive assessment of endurance capabilities within the constraints of large-scale recruitment procedures. Further exploration into incorporating other relevant physiological metrics, such as heart rate recovery or lactate threshold, alongside data from these alternative field tests, could further enhance the accuracy of predicting 12-min run times. This multi-faceted approach holds the potential to provide a more comprehensive assessment of endurance capabilities within the constraints of large-scale recruitment procedures. Ultimately, investing in research to refine assessment strategies and ensure a highly capable and prepared force is paramount for the Czech Army.

This study, while providing valuable insights into the relationship between PWC 170 and 12-min run performance in a specific cohort of Czech military personnel, acknowledges the need for further research to enhance its novelty and broaden its implications. As suggested by the reviewer, future studies should aim for a larger and more representative sample encompassing various military roles and experience levels. Furthermore, incorporating additional physiological metrics such as body composition analysis, heart rate recovery, and lactate threshold, alongside data from field-based assessments, could lead to the development of more robust and comprehensive predictive models for endurance performance. Such models would be invaluable for optimizing recruitment procedures and tailoring training programs within a military context.

## Data Availability

The raw data supporting the conclusions of this article will be made available by the authors without undue reservation.
